# CSCs in Breast Cancer—One Size Does Not Fit All: Therapeutic Advances in Targeting Heterogeneous Epithelial and Mesenchymal CSCs

**DOI:** 10.3390/cancers11081128

**Published:** 2019-08-07

**Authors:** Andrew Sulaiman, Sarah McGarry, Xianghui Han, Sheng Liu, Lisheng Wang

**Affiliations:** 1 Department of Biochemistry, Microbiology and Immunology, Faculty of Medicine, University of Ottawa, 451 Smyth Road, Ottawa, ON K1H 8M5, Canada; 2 China-Canada Centre of Research for Digestive Diseases, University of Ottawa, 451 Smyth Road, Ottawa, ON K1H 8M5, Canada; 3 Institute of Digestive Diseases, Longhua Hospital, Shanghai University of Traditional Chinese Medicine, 725 South Wanping Road, Shanghai 200032, China; 4 Regenerative Medicine Program, Ottawa Hospital Research Institute, Ottawa, ON K1H 8L6, Canada; 5 Institute of Chinese Traditional Surgery, Longhua Hospital Affiliated to Shanghai University of Traditional Chinese Medicine, 725 Wanping South Road, Shanghai 200032, China; 6 Ottawa Institute of Systems Biology, University of Ottawa, 451 Smyth Road, Ottawa, ON K1H 8M5, Canada

**Keywords:** breast cancer, triple negative breast cancer, cancer stem cell, epithelial, mesenchymal, Wnt, YAP, NF-κB, hypoxia

## Abstract

Unlike other breast cancer subtypes, triple-negative breast cancer (TNBC) has no specific targets and is characterized as one of the most aggressive subtypes of breast cancer that disproportionately accounts for the majority of breast cancer-related deaths. Current conventional chemotherapeutics target the bulk tumor population, but not the cancer stem cells (CSCs) that are capable of initiating new tumors to cause disease relapse. Recent studies have identified distinct epithelial-like (E) ALDH^+^ CSCs, mesenchymal-like (M) CD44^+^/CD24^−^ CSCs, and hybrid E/M ALDH^+^/CD44^+^/CD24^−^ CSCs. These subtypes of CSCs exhibit differential signal pathway regulations, possess plasticity, and respond differently to treatment. As such, co-inhibition of different subtypes of CSCs is key to viable therapy. This review serves to highlight different pathway regulations in E and M CSCs in TNBC, and to further describe their role in disease progression. Potential inhibitors targeting E and/or M CSCs based on clinical trials are summarized for further investigation. Since future research needs to adopt suitable tumor models and take into account the divergence of E and M CSCs for the development of effective treatments, TNBC models for clinically translatable studies are further discussed.

## 1. Introduction 

Breast cancer is the most frequently diagnosed cancers among the female population, accounting for an incidence of one in four women worldwide [1]. Despite improvements in treatment, it remains the leading cause of cancer-related deaths among women [1]. Although triple negative breast cancer (TNBC) accounts for 15–20% of breast cancer incidence, this subtype is hard to treat and is disproportionally associated with the majority of breast cancer-related deaths [2].

Unlike the other breast cancer subtypes, triple negative breast cancer is negative for ER (Estrogen Receptor), PR (Progesterone Receptor), and HER-2 (human epidermal growth factor receptor 2) receptors. Non-specific chemotherapy remains as first-line treatment for TNBC due to the lack of specific targets. However, chemotherapy is associated with off-target toxicity and the enrichment of cancer stem cells (CSCs) [3,4,5]. This limitation, in combination with the aggressive nature of TNBC, may contribute to the poor prognosis of TNBC in comparison to other breast cancer subtypes. For women over 50 years of age, the five-year disease-free survival was 92.4~94.8% for patients with ER+ breast cancer, 82% for patients with HER-2+ breast cancer, and 53.3% for patients with TNBC [6].

A major challenge for effective treatment of TNBC is to eliminate CSCs. CSCs have the capacity to self-renew, differentiate, dedifferentiate, and hibernate [7], capable of initiating new tumors to cause disease relapse [8,9]. Although conventional chemotherapeutic drugs effectively suppress bulk tumor cells, they are ineffective in inhibiting CSCs, and even enrich CSCs following treatment [3,4,5]. CSCs in TNBC are heterogeneous, interconvertible, and respond to chemotherapy differently [10,11,12,13]. This review will discuss the main CSC subtypes, their relevance to TNBC disease progression, and clinical approaches for the potential treatment.

## 2. Overview of Epithelial and Mesenchymal CSCs in Breast Cancer

CSCs are a small population residing within tumors. They exhibit stem cell-like properties, such as self-renewal, differentiation, dedifferentiation, trans-differentiation, symmetric/asymmetric division, and quiescence [14]. CSCs have been found to exist in different types of solid tumors and are at the apex of the cellular hierarchy in tumors, capable of maintaining CSC pools and giving rise to non-CSC bulk tumor cells to promote disease progression [15]. 

Al Hajj et al. made the first demonstration that the fractionated CD44^+^/CD24^−^ subpopulation from breast cancer patients exhibited a 100-fold greater capacity to form tumors (i.e., tumorigenicity) compared to those unsorted cells after transplantation into mammary pad of immunodeficient mice [16]. CD44 (cluster of differentiation 44) is a class I transmembrane glycoprotein which acts as a receptor for hyaluronic acid and is associated with modulating mesenchymal-like processes such as cell adhesion, invasion, and migration [17,18]. In contrast, CD24 (cluster of differentiation 24) is associated with carbohydrate metabolism and epithelial-like breast cancer cells [19]. CD44^+^/CD24^−^ CSCs are associated with a mesenchymal-like phenotype that is highly metastatic/invasive and possesses a greater tumorigenesis capacity [18]. 

Another marker for breast CSCs is aldehyde dehydrogenases (ALDH), which is frequently used as a marker for hematopoietic stem cells [20]. ALDH are comprised of 19 isomers which catalyze the oxidation of aldehydes and convert aldehydes to carboxylic acids, important for cellular detoxification after exposure to chemotherapeutic agents (e.g., cyclophosphamide) [21]. High ALDH expression has been associated with CSCs in a wide variety of cancers, including breast cancer [21]. The fractionated ALDH^+^ cells from patients with breast tumor possessed greater tumorigenic potentials, regenerating new tumors with as few as 1500 cells. In contrast, ALDH^−^ cells were not capable of forming tumors even after injection of 50,000 cells [22]. Interestingly, ALDH is predominantly expressed in epithelium tissues in the brain, liver, kidneys, and breast [21]. ALDH^+^ breast cancer CSCs exhibit an epithelial-like phenotype [11].

In a landmark study, Liu et al. reported that CD44^+^/CD24^−^ CSCs resided at the edge of the breast tumor with low expression of E-cadherin but high expression of vimentin and ZEB1 (Zinc Finger E-Box Binding Homeobox 1), exhibiting a mesenchymal, migratory, and invasive phenotype [11]. In contrast, ALDH^+^ CSCs resided within the core of the tumor with high expression of E-cadherin but low expression of vimentin and ZEB1, exhibiting an epithelial phenotype [11]. This study demonstrated that M (mesenchymal, CD44^+^/CD24^−^) and E (epithelial, ALDH+) CSCs were distinct populations with different patterns in tumor distribution, gene expression, proliferation, and quiescence, suggesting different functionalities of these two CSC subpopulations [11]. E and M CSCs existed in all breast cancer subtypes, but their proportions were varied. Basal TNBC cell lines were enriched with both E and M CSCs compared to their luminal breast cancer counterparts [11]. 

E and M CSCs were found to be interconvertible. Liu et al. demonstrated that fractionated M or E CSCs from breast cancer cell lines gradually reconstituted the heterogeneous tumor population (bulk, CD44^+^/CD24^−^, and ALDH^+^ tumor cells) [11]. Together, these findings support the existence of distinct mesenchymal and epithelial CSCs within breast tumors, and demonstrate that these populations are plastic, capable of reconstituting other CSC and non-CSC populations [11]. 

Cancer metastasis is responsible for 90% of cancer-related deaths [23]. Epithelial to mesenchymal (EMT) transition is a biological process, which facilitates tumor cell dissociation, migration, and metastasis [24]. At the core of tumor, when cancer cells are undergoing EMT, epithelial cells gain a mesenchymal phenotype (e.g., loss of E-cadherin, cytokeratin, and claudin while acquiring N-cadherin and vimentin). This change reduced cell-cell adhesion but increased migration and invasion of cancer cells, moving from the tumor core to the edge [24]. M CSCs at the edge of the tumor invade the surrounding tissue, translocate into the bloodstream, and then migrate to different tissues [11,25]. Upon arriving at a suitable secondary location, the M CSCs convert into an E CSC state, capable of promoting angiogenesis and growing quickly in hypoxic conditions [25]. Conventionally, EMT is thought to facilitate metastasis, while MET is critical for secondary tumor formation. Within the secondary tumor, the E CSC population can differentiate into bulk tumor cells, self-renew to maintain its population, and convert into M CSCs to repeat the cycle [25].

Although the EMT/MET model for metastasis is well-studied and accepted to date, histological evidence in patient tumor samples has not been proved [26]. Furthermore, two recent studies have challenged the role of EMT/MET model in cancer metastasis [27,28]. Using a loss-of-function approach, Zheng et al. knocked out Twist or Snail (two critical EMT inducers) using Cre-recombinase in a pancreatic ductal adenocarcinoma mouse model in order to suppress EMT. However, the number of traced metastatic circulating tumor cells was not changed following Twist knockout. Additionally, metastatic tumor cells in various organs were negative for a mesenchymal marker (α-smooth muscle actin) compared to the controls, indicating that EMT was dispensable for metastasis [27]. 

Another study by Fischer et al. employed an EMT lineage-tracking system using a mesenchymal-specific promoter to track whether the metastatic lung cancer cells underwent EMT [28]. They found that breast cancer-lung metastasis maintained their epithelial phenotype, indicating that EMT was dispensable for metastasis [28].

There has been debate over the aforementioned studies [29,30]. For the Twist and Snail knockout studies used in the report of Zheng et al. [27], α-smooth muscle actin is not considered a reliable marker for EMT monitoring in the particular mouse model [29]. Additionally, after Twist and Snail knockout, unaltered metastasis of pancreatic ductal adenocarcinoma mouse cells may be due to the redundancies within the EMT process [29].

Another research group found that the Fsp1-cre transgene used in the manuscript of Fischer et al. may not be a critical modulator of EMT, as Fsp1 knockout mice are capable of undergoing all stages of EMT [30,31]. Fsp1 is also not expressed universally in carcinoma cells that have undergone EMT [30]. Additionally, the vimentin-Cre tracing marker used in Fischer et al. studies is only weakly expressed in carcinoma cells undergoing EMT, while tumor-associated stromal cells highly expressed vimentin, indicating a potential challenge for the lineage tracing system used [30]. 

These rebuttals highlight the complicated nature of EMT/MET in metastasis and secondary tumor formation. Fischer et al. replied to the rebuttal, defending their usage of vimentin and Fsp1 promoters as indicators of EMT. Additionally, Fischer et al. further demonstrated the fidelity and efficacy of the Fsp1-Cre RFP+ to GFP+ EMT model and showed that the GFP+ EMT cells constitute only 4.46  ±  1.0% of the total primary tumor cells in the Vimentin–Cre model. Importantly, none of the metastases observed were derived from these GFP+ EMT cells. Based on the specificity of their models, Fischer et al. argued that EMT is not required for metastasis [32]. 

**Figure 1:**
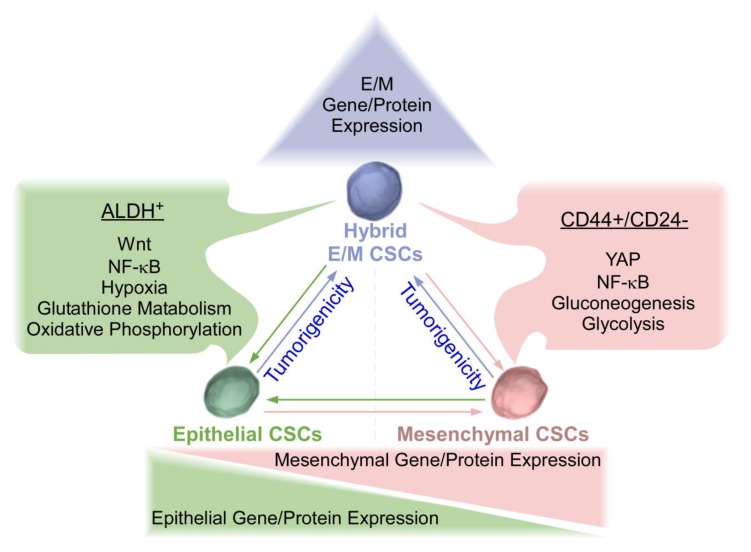
Epithelial, Mesenchymal, and Hybrid triple-negative breast cancer (TNBC) cancer stem cells (CSCs). TNBC mesenchymal (M) CSCs are characterized by CD44^+^/CD24^−^ with elevated levels of Yes associated protein (YAP), nuclear factor kappa-light-chain-enhancer of activated B cells (NF-κB), and enhanced gluconeogenesis and glycolysis. Conversely, TNBC epithelial (E) CSCs are characterized by ALDH^+^ with elevated levels of Wnt, NF-κB, hypoxia, enhanced glutathione metabolism, and oxidative phosphorylation. Epithelial and mesenchymal CSCs exhibited plasticity and were interconvertible. Recent studies have revealed that hybrid E/M CSCs are more tumorigenic than complete E and M counterparts and may be capable of differentiating into complete epithelial or mesenchymal CSCs. Plasticity amongst three states of CSCs needs to be considered for the development of effective therapeutic strategies for TNBC.

Indeed, increasing evidence supports that tumor cells do not need to undergo a complete EMT/MET shift for metastasis and formation of secondary tumors [25,33,34]. Additionally, the identification of CSCs expressing both M and E markers suggests the existence of a hybrid E/M CSC phenotype [11,18]. Hybrid CSCs are cells in the process of EMT/MET. This hybrid state may facilitate mobility, survival, and reconstitution of secondary tumor. Hybrid CSCs have been shown to possess greater tumorigenicity and metastatic potential in comparison with complete EMT or MET CSC counterparts (Figure 1) [22,25,34]. Of note, different CSC populations can interconvert, which may just represent different epigenetic states within the same clonal population, warranting further studies. Emerging single-cell technologies (e.g., single-cell RNA sequencing, single-nucleus RNA-sequencing) provide a new opportunity to profile individual cells within tumors and to study tumor heterogeneity and metastasis at single-cell resolution [35]. For example, single-nucleus sequencing of breast cancers revealed that copy number evolution occurred in short bursts early in tumor evolution, whereas point mutations evolved gradually over time to produce more extensive clonal diversity [36]. Harnessing the power of single-cell assessment will lead to great insights into the properties of EMT, MET, and hybrid CSCs.

Understanding of E and M CSCs are critical for identification of key targets to develop novel therapeutics for TNBC treatment. An effective therapy needs to inhibit both E and M CSC subpopulations to abolish reconstitution of CSC pool, owing to the plasticity of E and M CSCs. Thus, relevant pathways essential for the development and survival of TNBC CSCs will be discussed below, such as Wnt, YAP (Yes associated protein), NF-κB (nuclear factor kappa-light-chain-enhancer of activated B cells), and hypoxia. These pathways have been demonstrated to be highly expressed in TNBC compared to other breast cancer subtypes, highlighting their potential as specific targets for TNBC treatment [37,38,39]. Importantly, some inhibitors and drugs targeting Wnt, YAP, NF-κB, and hypoxia have been tested in clinical trials, exhibiting preclinical efficacy and patient tolerability. This review will focus on these four pathways. Other relevant pathways in TNBC have recently been reviewed elsewhere [40,41,42].

## 3. Wnt/β-Catenin Signaling in Breast Cancer and TNBC and Its Association with Epithelial CSCs

Wnt signaling pathways are characterized into the canonical or β-catenin dependent pathway, and the non-canonical or β-catenin independent pathway. More focus has been on the canonical pathway in the field. Wnt/β-catenin canonical signaling is a highly conserved developmental pathway. It regulates self-renewal of hematopoietic, intestinal, and embryonic stem cells [43]. Wnt signaling is also essential for self-renewal and differentiation of mammary stem cell/progenitor cells [44]. Dysregulated Wnt/β-catenin signaling has been shown to be highly expressed in TNBC and inversely correlated with poor patient survival, promoting CSC enrichment, chemoresistance, and metastasis. Wnt/β-catenin signaling is also associated with E CSC expansion [45,46,47,48,49]. Interestingly, Wnt/β-catenin mutations commonly found in other cancer types are rarely found in breast cancer [50]. 

Canonic Wnt activation contributes to chemoresistance, and chemotherapy enhances Wnt signaling in breast cancer [3]. It has been shown that WNT10B signal axis was elevated in TNBC and correlated with chemoresistance and poor patient prognosis [51]. HMGA2 (High Mobility Group AT-Hook 2, a direct target of WNT10B) is linked with EZH2 (Enhancer of Zeste 2, a methyltransferase which epigenetically modulates chromatin) activity, and directly interacted with β-catenin to stimulate Wnt activity [52,53]. The WNT10B/β-catenin/HMGA2/EZH2 cascade is expressed in TNBC and associated with increased rates of metastasis and decreased recurrence-free survival by 2.4-fold [51]. Ayachi et al. further revealed an autoregulatory loop between EZH2 and HMGA2 in TNBC cells [54]. HMGA-EZH2 protein-protein interactions were required for CBP (CREB-binding protein) K49 acetylation of β-catenin to modulate Wnt activity [54]. Moreover, both HMGA2/EZH2 promoted EMT in TNBC, and deletion of either one significantly reduced tumor growth and metastasis without affecting tumorigenicity [54]. Using TNBC patient derived xenografts (PDX), it was demonstrated that Wnt signaling was highly activated in both chemoresistant and naïve TNBC PDX tumors. Furthermore, all PDX tumors were sensitive to the treatment with small molecule inhibitor ICG-001 or PRI-724. ICG-001 and PRI-724 are distinct chemical entities, albeit both are CBP/β-catenin antagonists. Only PRI-724 has been tested in the clinic [54], and was demonstrated to be safe and tolerable to patients [55].

Wnt/β-catenin signaling has been associated with E CSCs. Wnt is more highly expressed in E ALDH^+^ CSC populations than in M CD44^+^/CD24^−^ CSCs [56,57]. Moreover, E CSCs are more sensitive to Wnt inhibition than M CSCs [56,58]. Domenici et al. recently found that Sox9 (Sry-related HMG box 9, an essential modulator of mammary gland development) transcription factor is a key modulator of self-renewal of breast luminal progenitor cells. The expression levels of Sox9 were found to be higher in breast tumors and the highest in TNBC samples, with the fractionated ALDH^+^ breast tumor cells possessing higher levels of Sox9 mRNA [59]. Sox9 is a poor prognostic indicator and is strongly associated with Wnt signaling via LRP6 (Low-density lipoprotein receptor-related protein 6) and TCF4 (Transcription Factor 4) in breast cancer [60]. Inhibition of Sox9 reduced tumorigenicity and E CSC population, and sensitized breast cancer cells to therapeutic intervention [59]. 

Interestingly, latency competent breast cancer cells (LCBCC, cancer cells that can reseed organs with latent metastasis) have been found to maintain a stem cell-like state and express Sox2 and Sox9 transcription factors. Surveillance of natural killer (NK) cells prevents these LCBCC from infiltrating into organs and expanding. However, the LCBCC cells can downregulate NK cell activators and develop resistance. LCBCC express high levels of DKK1 (Dickkopf-related protein 1) to inhibit Wnt signaling and enforce a quiescent state. Knockdown of DKK1 increases metastatic growth, thus linking Wnt signaling with long-term quiescence in disseminated cancer cells that cause latent metastasis and relapse [61]. This adds an additional layer of complexity to the role of canonical Wnt signaling in breast cancer CSCs. Furthermore, different from the role of canonical Wnt pathway in E CSCs, non-canonical Wnt signals have recently been shown to promote invasion, survival, and metastasis of CSCs, and is likely important in M phenotypic TNBC CSCs [49]. Further studies of the roles of canonical and non-canonical Wnt pathways in CSC are required.

Combination of canonical Wnt inhibitors with conventional chemotherapeutic agents seems to be an attractive approach for TNBC treatment. Synergistic reduction of tumor growth and metastasis has been observed when chemoresistant TNBC PDX tumors were treated with doxorubicin and the Wnt inhibitor ICG-001/PRI-724 [54,62]. Active and interventional clinical trials in Clinicaltrials.gov database for the treatment of patients with TNBC are summarized in Table 1. These potential Wnt modulators/inhibitors seem to be safe for the usage in clinic and have been demonstrated to suppress the Wnt signaling pathway in preclinical studies. Further studies will be needed to determine their clinical efficacy in combination with other inhibitors and chemotherapeutic drugs, as well as underlying mechanisms.

## 4. YAP Signaling in Breast Cancer and TNBC and Its Association with M CSCs

The Hippo-YAP pathway is a key developmental regulator of organ size, tissue growth, and stem cell maintenance [73,74]. This pathway is regulated by cell density, stiffness of the surrounding extracellular matrix, and nutrient availability [75]. Aberrant YAP expression has been associated with a wide myriad of cancer types, including breast cancer [76,77]. YAP overexpression promotes cancer cell proliferation, metastasis, chemoresistance, and tumorigenicity in breast cancer, and YAP was associated with M CSCs [76,78,79,80]. 

YAP has been shown to promote breast cancer stemness. YAP overexpression led to the upregulation of CSC-associated genes and IL-6 (Interleukin 6) through SRF (Serum response factor) [39]. YAP/TAZ/IL-6/SRF were highly expressed in TNBC compared to luminal breast cancer subtypes and is associated with the enrichment of M CSCs [39]. 

TAZ (Tafazzin, a homolog to YAP) has also been implicated in CSC enrichment and associated with M CSC phenotype. Knockdown of TAZ led to reduction of YAP/TAZ target genes and tumorigenicity. The fractionated CD44^+^/CD24^−^ M CSCs expressed high levels of TAZ protein compared to other control cells. Using a doxycycline-inducible TAZ vector, non-CSC CD44^−^/CD24^−^ cells after TAZ overexpression showed a similar tumorsphere-forming capacity to M CD44^+^/CD24^−^ CSCs, suggesting an important role of YAP/TAZ in M CSCs [81]. In addition, TAZ was found to interact with Scribble (a gatekeeper of epithelial polarity), leading to its delocalization from the cell membrane and the loss of apicobasal polarity [81].

The role of YAP in breast cancer and M CSCs is also supported by the studies of YAP/TAZ upstream modulators, Kibra (WW domain-containing protein 1, WWC1) and SREBP (Sterol regulatory element-binding protein)/mevalonate. Kibra has been shown to suppress EMT in breast cancer. In TNBC patients, Kibra expression was found to be diminished compared to luminal breast cancer patients [82]. Another important YAP modulator is SREBP/mevalonate pathway. Geranylgeranly pyrophosphate is produced by the mevalonate pathway that enhances Rho GTPases and subsequently activates YAP. Inhibition of mevalonate pathway by statins suppresses YAP localization and transcription. Statins promote apoptosis, sensitize TNBC to chemotherapy, and inhibit M CSCs [83,84,85]. Since statins are FDA-approved drug used regularly in clinic, they may be repurposed as a tangible approach for the treatment of TNBC by inhibiting M CSCs.

YAP has also been linked with other signaling pathways. Wang et al. demonstrated that YAP transcriptionally activated Sox9 via TEAD1-mediating signaling and dual inhibition of Sox9, and YAP robustly suppressed the growth of esophageal squamous cell carcinoma [86]. Domenici et al. reported that Sox9 also upregulates Wnt signaling in breast cancer [59]. Other studies showed interactions between Wnt and YAP signaling. It was found that ROR1 (Receptor Tyrosine Kinase Like Orphan Receptor 1) was elevated in invasive ductal adenocarcinoma following chemotherapy [87]. Using breast cancer PDX models, paclitaxel treatment stimulated ROR1 expression that was associated with CSC enrichment, spheroid formation, tumor invasiveness, and tumorigenicity. Further analysis revealed that ROR1 expression was associated with increased YAP/TAZ activity, and ROR1^high^ breast cancer cells expressed genes associated with M CSC phenotypes, providing an additional link between YAP/TAZ and M CSCs. Wnt5a was found to activate YAP/TAZ through ROR1. Monoclonal antibody cirmtuzumab against ROR1 inhibited the Wnt5a-stimulated RhoA, YAP/TAZ in PDX breast cancer models, and subsequently reduced metastasis and tumorigenicity [87]. Samanta et al. also found that TAZ can be regulated by an mRNA-binding protein, and that this regulation involved the integration of Hippo and alternative WNT-signaling pathways in breast cancer CSCs [88]. Recent studies showed that M CD44^+^/CD24^−^ CSCs expressed high levels of YAP than E ALDH+ CSCs and are more sensitive to YAP inhibition [56,87]. 

Active and interventional clinical trials in Clinicaltrials.gov database for the treatment of patients with TNBC are summarized in Table 2. These potential YAP inhibitors seem to be safe for the usage in clinic and have been demonstrated to suppress YAP signaling pathway in preclinical studies. Further studies will be needed to determine their clinical efficacy in combination with other inhibitors and chemotherapeutic drugs, as well as underlying mechanisms.

## 5. NF-κB, Cytokines and the Tumor Microenvironment in M and E CSCs

NF-κB signaling affects immunity, inflammation, cell survival, and proliferation [95,96]. This pathway is in part regulated by growth factors, cytokines, infection, DNA damage, stress, and hypoxia reactive oxygen species [95,96,97,98].

Chemotherapeutic agents inhibit bulk tumor cells but also upregulate NF-κB signaling, resulting in chronic inflammation [99]. Chronic inflammation leads to the accumulation/polarization of tumor associated macrophages (TAMs, proinflammatory M1 TAMs and anti-inflammatory M2 TAMs). M2 TAMs promote chemoresistance and breast cancer progression and metastasis [100]. After withdrawal of chemotherapy (e.g., paclitaxel, fluorouracil, and doxorubicin), both NF-κB and Wnt signaling pathways in TNBC were activated, promoting the release of inflammatory cytokines (IL-6, IL-8, etc.). This forms an autocrine forward-feedback loop, where the cytokines interact with multiple downstream pathways such as JAK1/2, STAT3, and AP1, which in turn enhances Wnt and NF-κB signaling and leads to more cytokine secretion [101]. Additionally, various pathways, such as PI3K-Akt, Rho-GTPase, MAPK, and STAT3, are also activated to promotes cancer proliferation, metastasis, chemoresistance, and E/M CSC enrichment [101,102,103,104]. Of note, IL-6 and IL-8 are overexpressed in TNBC compared to other breast cancer subtypes [105,106]. Although the exact mechanism by which cytokine secretion promotes CSC enrichment remains incompletely understood, it has been shown that secreted IL-6 activates the JAK1-STAT3 signal cascade in TNBC and promotes *OCT-4* gene expression in non-CSCs to convert non-CSCs into E/M CSCs [104]. This may partially explain why TNBC tumors after chemotherapy are highly tumorigenic compared to untreated controls [5,107]. Radiotherapy and surgery have also been shown to enhance NF-κB signaling to fuel the growth and metastasis of residual tumor cells via an autocrine feedback loop [108,109]. 

CSCs in breast cancer patients also exhibit higher levels of NF-κB signaling [110]. Yamamoto et al. demonstrated a strong correlation between NF-κB activation and E and M CSC enrichment in basal-like breast cancer [110]. Luminal-like breast cancer, however, did not show this correlation, suggesting that NF-κB may be a target for both E and M CSCs in TNBC [110]. NF-κB signaling in non-CSCs upregulates Notch signaling in CSCs to stimulate CSC expansion [110]. These findings suggest that inhibition of NF-κB produced by both CSCs and non-CSCs is required to control CSC pools.

NF-κB is also a potent regulator of adaptive immunity to control T and B lymphocyte development and function [108,111,112]. In cancers, NF-κB activation in T cell subpopulations is essential for an anti-tumor response [113,114,115]. Increased NF-κB signaling in CD4+ T cells has been linked with increased anti-tumor response by increased secretion of granzyme B, TNF-α (tumor necrosis factor alpha), and interferon-γ [116,117,118]. However, NF-κB also plays a major role in CD4+ Treg (T regulatory cells) activation, an immunosuppressive T cell population that supports tumor immune evasion and disease progression [33]. Treg cells migrate into the tumor microenvironment where they secrete anti-inflammatory cytokines, inhibit CD4+ T cell proliferation/function, and suppress anti-tumor immune response [119]. Ablation of NF-κB leads to reduced CD4+Foxp3+ Treg [120,121]. As such, NF-κB plays both immune activating and suppressing roles. Inhibition of this pathway should approached with caution in cancer immune therapy [122]. 

NF-κB has also been associated with Wnt, YAP, HIF (Hypoxia-Inducible Factors, described below), and others [123,124,125,126]. Recently, Wang et al. demonstrated that NF-κB upregulated YAP [127], and YAP also reciprocally activates NF-κB to enhance proinflammatory signaling [127]. Thus, inhibition of NF-κB has been considered for the treatment of TNBC [108]. 

Active and interventional clinical trials in Clinicaltrials.gov database for the treatment of patients with TNBC are summarized in Table 3. These potential NF-κB inhibitors seem to be safe for the usage in clinic and have been demonstrated to suppress the NF-κB signaling pathway in considerable preclinical studies. Further studies will be needed to determine their clinical efficacy in combination with other inhibitors and chemotherapeutic drugs.

## 6. Hypoxia Signaling in Breast Cancer and TNBC E CSCs

Hypoxia has been considered a key component in cancer progression, chemoresistance, and metastasis. The cells within tumor center undergo necrosis due to poor blood supply and low oxygen. However, cells in-between oxygenation and necrosis areas are alive under hypoxic conditions. These cells are relatively resistant to chemotherapy and radiotherapy, which are associated with cancer cell senescence, low drug concentration, and differential metabolic and signaling patterns [138,139,140,141]. A transcriptional factor predominantly detected in hypoxic tissues of TNBC is HIF-1α [142]. Hypoxia promotes angiogenesis, glycolysis, EMT/MET, E CSC enrichment, and tumorigenicity [143,144,145,146,147]. Hypoxia has also been linked with immune evasion. HIF-1α upregulates CD47 expression in breast cancer to enables evasion of macrophages [148].

Elevated HIF-1α activity was observed in the ALDH+ E CSC population of cultured human breast cancer cells. When ALDH-negative populations were exposed to hypoxic conditions, ALDH activity was increased, promoting E CSC enrichment. This observation supports the notion that hypoxia influences E CSC fate [149].

HIF-1α may partially account for the metastatic, invasive, and chemoresistant particularities of TNBC compared to the other breast cancer subtypes. In TNBC, chemotherapeutic agents such as paclitaxel stimulate HIF-1α, which promotes the secretion of IL-6, IL-8, and MDR-1 (Multidrug resistance protein 1) to enhance chemotherapeutic drug efflux, CSC resistance, tumorigenesis and E CSC enrichment [150,151,152]. Moreover, HIF-1α-mediated MDR-1 expression and chemoresistance was increased in the ALDH^+^ E CSC population [150]. 

Using a 13-gene hypoxia signature (*RRAGD*, *FABP5*, *UCHL1*, *GAL*, *PLOD*, *DDIT4*, *VEGF*, *ADM*, *ANGPTL4*, *NDRG1*, *NP*, *SLC16A3*, and *C14ORF58*), Perou et al. found that basal-like breast cancer and claudin-low TNBC samples expressed the highest levels of hypoxia genes compared to luminal or HER-2 positive breast cancer subtypes, suggesting that hypoxia inhibition could be one of strategies for TNBC treatment [153]. 

VEGF (vascular endothelial growth factor) is a downstream target gene of HIF-1α transcription. VEGF is positively correlated with E CSC enrichment and negatively correlated with poor prognosis of the patients with breast cancer [154]. VEGF co-receptor Neuropilin 1 (NRP-1) is also elevated in TNBC compared to luminal breast cancer and is enriched in E CSCs [155]. Further investigation revealed that the VEGF/NRP-1 axis promoted CSC enrichment in TNBC via upregulation of the Wnt/β-catenin pathway [155].

Exposure of TNBC cells to VEGF upregulated VEGFR-2 (one of VEGF receptors) and increased E CSCs, spheroid formation, metastasis, and tumor-forming capabilities [154]. Mechanistically, VEGFR-2 interacts with JAK2/STAT3 pathway to allow STAT3 to bind to the promoters of *SOX2* and *MYC* and induce the expression of these two CSC-associated genes, subsequently promoting CSC enrichment [154]. Of note, in luminal breast cancer, VEGF exposure was found to neither affect STAT3, *SOX2*, and *MYC* levels, nor CSCs [154].

Together, the association between TNBC, ALDH, VEGF/VEGFR-2, and HIF-1α makes targeting the hypoxia pathway of particular interest, which may lead to the development of a combinational strategy to suppress E CSCs to improve the treatment efficacy of TNBC [131,142,156,157]. Active and interventional clinical trials in Clinicaltrials.gov database for the treatment of patients with TNBC are summarized in Table 4. These potential hypoxia inhibitors seem to be safe for the usage in clinic and have been demonstrated to suppress the hypoxia signaling pathway in preclinical studies.

## 7. Future Directions

Combination use of the aforementioned pathway inhibitors with other conventional treatments may lead to breakthroughs in TNBC therapy in the near future. As chemotherapies remain the frontline treatment for TNBC patients and effectively reduce tumor burden, reducing/preventing/killing chemotherapy-induced M and E CSCs should be a focus for preclinical research and clinical translation.

Choosing the right animal models for in vivo studies is crucial for clinical translation. Although breast cancer cell lines have been wildly used and have provided important biological insights, they do not fully represent original tumors. TNBC cell lines are originally isolated from patient tumors. They are subsequently cultured in vitro for a prolonged period and selected for a cell clone adapted to immortally grow in two-dimensional and artificial conditions. TNBC cell lines do not reflect original tumor heterogeneous, three-dimensional structure, vascularity, and tumor microenvironment (e.g., extracellular matrix, infiltrating immune cells, and stromal populations) [167,168,169,170].

Additionally, in vitro culture conditions and selective procedures lead to the accumulation of mutations and altered gene expression, which can generate variants even from the same cell line. Remarkable discrepancies between the results generated from cancer cell lines and clinical trials have been observed [168,171,172,173]. A recent example is NCTO2399137, a Phase 2 study of MM-141 in combination with nab-paclitaxel and gemcitabine in metastatic pancreatic cancer (CARRIE) [174]. This trial was originally developed from several preclinical experiments using cancer cell lines that clearly demonstrated high efficacy of MM-141 in the treatment of pancreatic cancer [175,176,177], but it did not meet primary or secondary efficacy endpoints in patients compared to the chemotherapeutic agents, leading to the cessation of MM-141 development. Such frequent disparities between bench and bedside have led the U.S. National Cancer Institute to retire its panel of 60 human cancer cell lines for drug-screening since 2016 [178].

Of note, it has been shown that M and E CSCs are spatially separated in in vivo breast cancer tumors [10,11]. One hypothesis behind this observation is that the tumor microenvironment (TME) may play an essential role in tumor survival and spatially separation of the M and E CSC populations. Thus, targeting TME has been considered as one of the effective approaches for cancer treatment. It has been found that hypoxic regions of TME promote CSC enrichment via upregulation of HIF-1a activity, and TME stiffness affects YAP signaling of tumor cells [179,180]. In addition, mathematical modeling demonstrated that spatial gradients of EMT-inducing molecules, such as transforming growth factor β (TGF-β) from the tumor stromal boundary, promoted Notch-Jagged/Notch-Delta signaling to control CSC development, with M CSCs on the edge of the tumor (being exposed to high levels of TGF-β and EMT signals) and E or hybrid CSCs on the interior (being exposed to less TGF-β and EMT signals) [10]. Although TME is important for the regulation, maintenance, and distribution of CSCs, it is absent in in vitro culture systems.

A clinically translatable model is required for drug-screening. PDX models are generated by patient tumor fragments directly implanted into immunocompromised mice [168,170]. Tumor structure, heterogeneity, vasculature, and tumor microenvironment are preserved. Thus, PDX models better resemble primary patient tumors, exhibiting high clinical concordance (92%) [168,181,182]. Furthermore, “humanized” mouse models have been created by engraftment of CD34+ human blood stem/progenitor cells to generate human immune system, followed by implantation of PDX tumors [183,184]. Engrafting TNBC PDX tumors into this model allows for the study of immunotherapies (anti-PD-1, CAR-T, etc.) or therapeutic modulation of the immune system [185,186]. 

PDX preclinical models are increasingly considered as a powerful system for clinically translational studies. These models can be used to study E/M CSC plasticity and biology. A report by Luo et al. investigated E/M CSC plasticity in TNBC and found that suppression of glycolysis effectively inhibited M CSCs but increased the E CSCs. Further investigation revealed that glycolytic inhibition promoted oxidative stress to fuel the transition from M to E CSCs [187]. This process, however, was sensitive to antioxidant N-acetylcysteine. Using TNBC PDX models, it was found that E and M CSCs were driven by distinct metabolic pathways. E CSCs relied more on OXPHOS, glutathione metabolism, and cell cycle signaling, while M CSCs relied more on glycolysis and gluconeogenesis [187]. Both E/M CSCs shared cytokine-cytokine receptor interaction signaling [187].

Further investigation revealed that AMPK-HIF-1α axis was critical to M to E CSC plasticity. Inhibition of glycolysis stimulated HIF-1α protein expression and AMPK signaling. Treatment with N-acetylcysteine disrupted this process, suggesting that increased reactive oxygen species (ROS) via AMPK activation stabilized HIF-1α to mediate M to E CSC plasticity/transition. TNBC PDX models revealed that NRF2 (NF-E2-related factor 2) antioxidant activity is critical for E CSCs to cope with higher ROS levels and survive under hypoxia. Moreover, inhibition of antioxidant signaling downstream of NRF2 was found to inhibit E CSCs. Co-inhibition of glycolysis and antioxidant pathways was capable of effectively inhibiting both E and M CSCs in a PDX model [187]. 

## 8. Conclusions

Since E and M CSC populations are regulated by different signaling pathways, possess plasticity, and respond differently to treatment, dual inhibition of E and M CSCs seems to be essential for the effective treatment of TNBC and other subtypes of breast cancers. To develop feasible and effective therapeutics, future preclinical research should consider both E and M CSCs and assess how experimental treatments affect these populations using clinically translatable models. Currently, therapeutic approaches remain elusive, with no FDA-approved specific Wnt, YAP/TAZ, or NF-κB inhibitors. Given the importance of those pathways in development and homeostasis of normal tissues/cells, complete inhibition may cause side effects or toxicities. Harnessing the power of single-cell assessment may provide great mechanistic insights and reveal specific targets for EMT, MET, and hybrid CSCs. With new discoveries, further studying the current Wnt, YAP/TAZ, NF-κB, and hypoxia inhibitors tested in clinical trials and searching for new inhibitors may lead to breakthrough.

## Figures and Tables

**Table 1:** Potential Wnt Inhibitors Tested in TNBC Clinical Trials.

Inhibitor	Clinical Trial Number	Mechanism	References
RX-5902	NCT02003092	Inhibits phosphorylated p68 RNA helicase preventing nuclear β-catenin translocation and Wnt signaling	[63,64]
CB-839	NCT03057600	Glutaminase Inhibitor (GSL1). GLS1 has been found to promote stemness via reactive oxygen species/Wnt/ β-catenin signaling	[65]
Eribulin mesylate	NCT02513472	Inhibitor of microtubule dynamics and demonstrated Wnt-related gene suppressive properties	[66]
Selinexor	NCT02402764	Selective inhibitor of nuclear export (SINE) that blocks XPO1 leading to forced nuclear retention of major tumor suppressor proteins reducing β-catenin	[67]
Sorafenib	NCT02624700	Tyrosine protein kinase inhibitor and reduces β-catenin and Wnt signaling	[68]
Cetuximab	NCT01097642	Monoclonal antibody which binds to and inhibits EGFR. Also Inhibits of MAPK which leads to inhibition of β-catenin nuclear activity.	[69]
Indomethacin	NCT02950259	Nonsteroidal anti-inflammatory drug which inhibits prostaglandins which is capable of suppressing β-catenin expression.	[70]
Bicalutamide	NCT03090165	Androgen antagonist preventing Wnt/β-catenin signaling	[71]

Note: The Clinicaltrials.gov database was used to assess active, interventional clinical trials for TNBC treatment within phase 1, 2, 3, or 4 of development. Following inhibitor identification, literature was consulted to determine any Wnt modulating effects [72].

**Table 2:** Potential YAP Inhibitors Tested in Active TNBC Clinical Trials.

Inhibitor	Clinical Trial Number	Mechanism	References
Zoledronic Acid	NCT02595138	Bisphosphonate which inhibits bone resorption and also inhibits farnesyl diphosphate synthase	[89]
Erlotinib	NCT02071862	Epidermal growth factor receptor (EGFR) Inhibitor which can sequester YAP in the cytoplasm	[90]
Trametinib	NCT01964924	MEK1/2 Inhibitor leading to decreased YAP protein levels and transcriptional activity.	[91]
Indomethacin	NCT02950259	Nonsteroidal anti-inflammatory drug that inhibits prostaglandins and is associated with YAP1 stimulation.	[92]
Selumetinib (AZD6244)	NCT02583542	MEK1/2 inhibitor which reduces YAP protein levels	[92]
Ipatasertib	NCT02162719	ATP-competitive, selective AKT inhibitor which can reverse EMT conferred by YAP overexpression	[93]
Alisertib (MLN8237)	NCT02187991	Aurora kinase A inhibitor which was capable of suppressing YAP protein levels	[94]

Note: The Clinicaltrials.gov database was used to assess active, interventional clinical trials for TNBC treatment within phase 1, 2, 3, or 4 of development. Following inhibitor identification, literature was consulted to determine any YAP modulating effects [72].

**Table 3:** Potential NF-κB Inhibitors in Active TNBC Clinical Trials.

Inhibitor	Clinical Trial Number	Mechanism of Action	References
Ribociclib	NCT03090165	CDK6 inhibition which prevents CDK6 phosphorylation and activation of NF-κB	[128,129]
Veliparib	NCT02032277	PARP1 and PARP2 inhibitor preventing PARP1 induced NF-κB activity and IL-6/STAT3 expression	[130]
Selinexor	NCT02402764	Selective inhibitor of nuclear export (SINE) that specifically blocks XPO1 leading to forced nuclear retention of major tumor suppressor proteins (TSPs) and inhibits NF-κB transcription.	[131]
Reparixin	NCT02370238	IL8 receptor CXCR1/2 inhibitor	[3]
Olaparib	NCT01116648	PARP Inhibitor which modulates PAR–p53–NF-κB activity	[132]
Omeprazol	NCT02950259	Proton pump inhibitor which interferes with NF-κB activation	[133,134]
CUDC-907	NCT02307240	PI3K/HDAC inhibitor which was demonstrated to inhibit NF-κB via stimulation IkBα and down-regulation of IKK beta and IRF4	[135]
Entinostat	NCT02708680	class I HDAC inhibitor which inhibits NF-κB, IL-6 and IL-8 gene signaling	[136]
Azacitidine	NCT01349959	DNA methyltransferase inhibitor, Inhibits IL-6 and NF-κB nuclear translocation	[137]

Note: The Clinicaltrials.gov database was used to assess active, interventional clinical trials for TNBC treatment within phase 1, 2, 3, or 4 of development. Following inhibitor identification, literature was consulted to determine any NF-κB modulating effects [72].

**Table 4:** Potential Hypoxia Inhibitors Used in Active TNBC Clinical Trials.

Inhibitor	Clinical Trial Number	Mechanism of Action	References
Bicalutamide	NCT03090165	Androgen antagonist preventing AR-induced hypoxia signaling	[158,159]
Zoledronic Acid	NCT02595138	Bisphosphonate which inhibits bone resorption and also inhibits HIF-1α transcription via inhibition of RAS/MAPK/ERK1/2	[160]
Eribulin mesylate	NCT02513472	Inhibitor of microtubule dynamics and can induce tumor vascular remodeling, reducing hypoxia	[66]
Everolimus	NCT01931163	Rapamycin derivative, mTORC1 inhibitor which reduces HIF-1α expression	[161]
Sorafenib	NCT02624700	Tyrosine protein kinase inhibitor and mediated inhibition of HIF-1a and VEGF proteins via modulation of mTOR/p70S6K/4E-BP1 and ERK phosphorylation.	[162]
Cetuximab	NCT01097642	Monoclonal antibody which binds to and inhibits EGFR and down-regulates HIF-1α levels by inhibiting synthesis of HIF-1α.	[163]
Trametinib	NCT01964924	MEK1/2 Inhibitor leading to the inhibition of HIF-1a transcriptional activity	[164]
BKM120	NCT02000882	P13K/Akt inhibitor which increases mitochondrial oxygen consumption and inhibits hypoxia	[165]
Selumetinib (AZD6244)	NCT02583542	MEK1/2 inhibitor which reduces HIF-1a activity.	[166]
Entinostat	NCT02708680	Class I HDAC inhibitor which inhibits HIF-1α gene signaling	[136]

Note: The Clinicaltrials.gov database was used to assess active, interventional clinical trials for TNBC treatment within phase 1, 2, 3, or 4 of development. Following inhibitor identification, literature was consulted to determine any hypoxia modulating effects [72].

## Abbreviations

ALDH	aldehyde dehydrogenase
CD44	cluster of differentiation 44 (hyaluronic acid receptor)
CD24	cluster of differentiation 24
CSC	cancer stem cell
PDX	patient derived xenograft
TNBC	triple negative breast cancer
